# In-hospital mortality in patients with acute limb ischemia over a 12-year period in the Brazilian public health-care system

**DOI:** 10.1590/1677-5449.210107

**Published:** 2022-01-07

**Authors:** João Henrique Fonseca do Nascimento, André Gusmão Cunha, André Bouzas de Andrade, Monique Magnavita Borba da Fonseca Cerqueira

**Affiliations:** 1 Universidade do Estado da Bahia – UNEB, Departamento de Ciências da Vida, Salvador, BA, Brasil.

**Keywords:** peripheral arterial diseases, arterial obstructive diseases, emergencies, gender, in-hospital mortality, doença arterial periférica, arteriopatias oclusivas, emergência, gênero, mortalidade hospitalar

## Abstract

**Background:**

Arterial diseases represent a severe public health problem in the 21st century. Although men have a higher overall prevalence, reports have suggested that women may exhibit atypical manifestations, be asymptomatic, and have hormonal peculiarities, resulting in worse outcomes and severe emergencies, such as acute limb ischemia (ALI).

**Objectives:**

To analyze the morbidity and mortality profile of ALI emergencies in Brazil between 2008 and 2019.

**Methods:**

An ecological study was carried out with secondary data from SIH/SUS, using ICD-10 code I.74 The proportions of emergency hospital admissions and in-hospital mortality rates (HMR) by gender, ethnicity, and age were extracted from the overall figures. P<0.05 was considered significant.

**Results:**

From 2008 to 2019, there were 195,567 urgent hospitalizations due to ALI in Brazil, 111,145 (56.8%) of which were of men. Women had a higher HMR (112:1,000 hospitalizations) than men (85:1,000 hospitalizations) (p<0.05), and a higher chance of death (OR=1.36; p<0.05). Furthermore, mean survival was significantly higher among men (8,483/year versus 6,254/year; p<0.05). Stratified by ethnicity, women who self-identified as white (OR=1.44; p<0.05), black (OR=1.33; p<0.05), and brown (RR=1.25; p <0.05) had greater chances of death than men in the same ethnicity categories. Moreover, women over the age of 50 years had a higher chance of death, with a progressive increment in risk as age increased.

**Conclusions:**

There was a trend to worse prognosis in ALI emergencies associated with women, especially in older groups. The literature shows that the reasons for these differences are still poorly investigated and more robust studies of this relevant disease in the area of vascular surgery are encouraged.

## Resumo

**Contexto:**

Doenças arteriais representam um grave problema de saúde pública no século XXI. Apesar de homens apresentarem maior prevalência geral, estudos sugerem que mulheres podem cursar com quadros assintomáticos, clínica atípica e particularidades hormonais, que resultam em desfechos desfavoráveis e urgências graves, como oclusões arteriais aguda (OAA).

**Objetivos:**

Analisar o perfil de morbimortalidade das urgências em OAA no Brasil entre 2008 e 2019.

**Métodos:**

Realizou-se estudo ecológico com dados secundários do Sistema de Informações Hospitalares/Sistema Único de Saúde, utilizando-se o código I.74 do Código Internacional de Doenças-10. Dos números absolutos, obteve-se proporções de internamentos de urgência e taxa de mortalidade intra-hospitalar (TMH) por gênero, etnia e idade. Considerou-se p < 0,05 significativo.

**Resultados:**

Entre 2008 e 2019, houve 195.567 internamentos de urgência por OAA no Brasil, dos quais 111.145 (56,8%) eram homens. Mulheres tiveram maior TMH (112:1.000 hospitalizações) em comparação a homens (85:1.000 hospitalizações) (p < 0,05), assim como maior chance de morte (*odds ratio* [OR] = 1,36; p < 0,05). Ademais, a média de sobrevida anual foi maior entre homens do que entre mulheres (8.483/ano vs. 6.254/ano, respectivamente; p < 0,05). Estratificando por etnia, mulheres apresentaram maior chance de óbitos entre brancas (OR = 1,44; p < 0,05), pretas (OR = 1,33; p < 0,05) e pardas (razão de risco [RR] = 1,25; p < 0,05), comparadas a homens das mesmas etnias. Nas análises etárias, mulheres com mais de 50 anos apresentaram maior chance de óbito, com aumento progressivo do risco com o envelhecimento.

**Conclusões:**

Nossas análises comparativas evidenciaram tendência de pior prognóstico nas urgências em OAA associadas a mulheres, sobretudo em grupos de idade avançada. A literatura evidencia que as razões para essas diferenças ainda são pouco estudadas, estimulando investigações mais robustas sobre essa importante casuística da cirurgia vascular.

## INTRODUCTION

Acute limb ischemia (ALI) represents one of the most common emergencies in vascular surgery, with incidence of approximately 1.5 per 10,000 persons per year.^1^^,^^2^ It is characterized by a sudden decrease in arterial perfusion of the limb, with a potential threat to the survival of the affected extremity, requiring urgent evaluation and management.^3^^-^5 ALI figures as a clinical emergency with eventual life-threatening outcomes and two of the most common etiologies are arterial embolism and *in situ* thrombosis of an atherosclerotic artery.^5^^-^7

Clinical events that cause ALI include embolism, acute thrombosis, dissection, or trauma.^1^^,^^2^ Acute thrombosis of an artery occurs most frequently at the site of an atherosclerotic plaque due to peripheral arterial disease (PAD), which is the designation of preference for partial or complete occlusions of the abdominal aorta or limb arteries.^1^^-^5

Historically, men were believed to have higher overall prevalence of cardiovascular diseases compared to women.^8^ However, recent studies have been assessing gender-differences for several arterial disorders and new premises have been posing female sex as an important risk factor for worse outcomes.^2^^,^^3^^,^^9^^,^^10^ Nonetheless, there is a lack of studies assessing gender and peripheral arterial obstruction emergencies in Brazil. Besides, there is concern about the relevant association between female sex hormones, the susceptibility to arterial diseases, and the differences in the periods of peaks and nadir of hormone levels during a lifetime, such as menacme and menopause.^11^^,^^12^ The mean age for menopause in Brazil is 51.2 years^13^ and postmenopausal women are at greater risk of PAD and ALI.^11^

Therefore, given the public health relevance of atherosclerotic arteriopathy and the scarcity of studies comparing sex-related outcomes and peripheral arterial disease in Brazil, this study aimed to evaluate gender disparities in hospital morbidity and mortality in emergency admissions for arterial thromboses and embolisms in the country’s Unified Health System (*Sistema Único de Saúde* – SUS).

## METHODS

This population-based, retrospective, and observational study, carried out with secondary data from a government database, evaluated gender as a specific risk factor for in-hospital morbidity and mortality related to emergencies due to arterial thromboses and embolisms in Brazil. The Unified Health System Department of Informatics (*Departamento de Informática do Sistema Único de Saúde* – DATASUS) is a publicly available and online platform, managed by the Ministry of Health (available for online access at http://tabnet.datasus.gov.br/cgi/deftohtm.exe?sih/cnv/nruf.def). Approval by a Research Ethics Committee is considered unnecessary because the secondary data employed were obtained from a public domain online database, without identification of patients, as stated by the National Commission of Research Ethics in Brazil (available at http://conselho.saude.gov.br/web_comissoes/conep/index.html).

The data were collected through the Hospital Information Systems (*Sistema de Informação Hospitalares em Morbidade Hospitalar* – SIH) from DATASUS, which gathers most of the information regarding hospital admission authorization forms, length of stay, diseases, and patient outcomes. The DATASUS platform defines *hospital admission* for an inpatient as remaining in hospital for more than 24 hours, thus outpatient contacts were not included in the analyses. All patient information was stratified geographically by place of residence.

Data were collected based on the International Disease Classification (10th Revision – ICD-10), using the I.74 code, recorded on DATASUS as “Arterial Thromboses and Embolisms” (ATE), for peripheral arteries. Therefore, events coded as ischemic stroke or myocardial infarction were not included. The period of study spanned from January 2008 to December 2019. To perform this investigation, we analyzed the following variables: gender, self-reported ethnicity (white, black, brown, or indigenous Brazilians), age (divided into and analyzed in five-year age groups), the total number of hospital admissions and the number of admissions by gender, the total number of in-hospital deaths and the in-hospital mortality rate, and the average length of stay. All searches run on the DATASUS' database were restricted to urgent hospital admissions only.

The normality of the distribution of variables was assessed using the Shapiro-Wilk test and Q-Q plots. In addition to testing the normality of the distribution, the homogeneity of the groups was assessed with Levene’s Test for Equality of Variances. Descriptive statistics such as mean, standard deviation (SD), median, and interquartile range (IQR: Q1-Q3), in addition to odds ratio (OR) and confidence interval (CI), were used to describe counts and proportions in the data.

Fisher’s exact test and the chi-square test with Yates’ continuity correction were used to compare proportions between two groups. Depending on the normality of the distribution of variables, the Mann-Whitney U test or Student’s *t* test for independent samples were also used when appropriate to compare differences between groups. Adjusted r^2^ values were obtained using linear regression to evaluate the variation of trends. P<0.05 results were considered significant.

The statistical analysis was conducted using BioEstat (*Instituto de Desenvolvimento Sustentável Mamirauá*, v. 5.3) and R software (RStudio, Inc. - R foundation for statistical computing, v. 4.0.3).

## RESULTS

**Figure 1:**
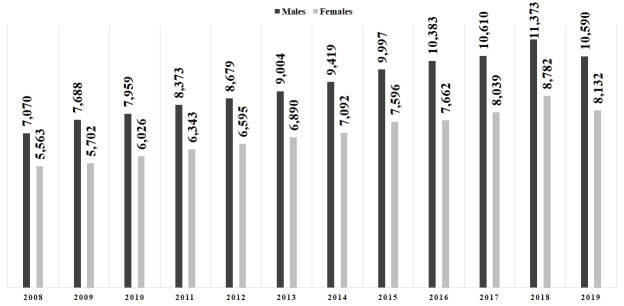
Total number of hospital admissions per year in 195,567 urgent hospitalizations due to (I.74) Arterial Thromboses and Embolisms in the Unified Health System in Brazil, by gender (2008-2019).

**Figure 2:**
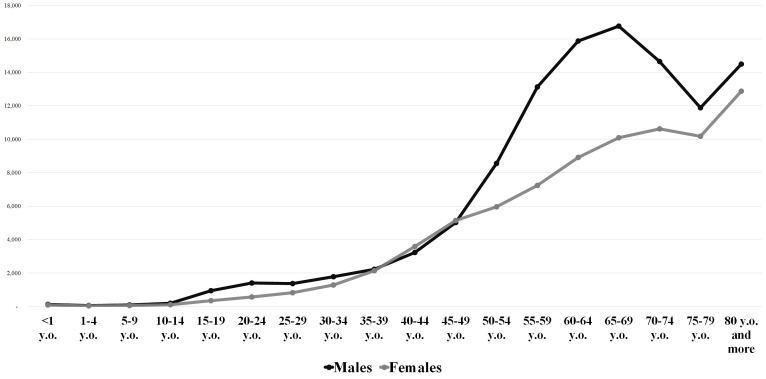
Age distribution of the total number of hospital admissions in 195,567 urgent hospitalizations due to (I.74) Arterial Thromboses and Embolisms in the Unified Health System in Brazil, by gender (2008-2019) (y. o.: years old).

There were 195,567 urgent hospital admissions due to ATE from 2008 to 2019, with an average of 16,297 cases/year (SD=2,273.16) (Table 1). Men accounted for 111,145 (56.8%) hospitalizations, with a mean of 9,262 cases/year (SD=1,292.14), whereas women totaled 84,422 (43.2%) admissions, with a mean of 7,035 cases/year (SD=984.45) (p<0.05) (Table 1; Figure 1). The average male/female ratio was 1.3/1. The mean age was 64.5 years (SD=16.1) for males and 65.5 years (SD=16.6) for females, but the peak age at hospital admission ranged from 65 to 69 years among men and 80 or more among women (Figure 2). The overall number of hospitalizations exhibited a rising trend over the years 2008 to 2019 (r^2^=0.9584), also observed in both males (r^2^=0.9554) and females (r^2^=0.959), with average annual growth rates of 3.8% (SD=0.04) and 3.6% (SD=0.04), respectively. There was no difference in the length of stay between men and women (8.7 days versus 8.6 days; p=0.95)

**Table 1:** Number of hospital admissions and in-hospital deaths in 195,567 urgent hospitalizations due to (I.74) Arterial Thromboses and Embolisms in the Unified Health System in Brazil, by gender (2008-2019).

	**HOSPITAL ADMISSIONS**			**IN-HOSPITAL DEATHS**		
	**OVERALL**	**MALE**	**FEMALE**	**OVERALL**	**MALE**	**FEMALE**
**2008**	12,633	7,070	5,563	1,231	607	624
**2009**	13,390	7,688	5,702	1,301	655	646
**2010**	13,985	7,959	6,026	1,447	724	723
**2011**	14,716	8,373	6,343	1,479	787	692
**2012**	15,274	8,679	6,595	1,550	785	765
**2013**	15,894	9,004	6,890	1,658	822	836
**2014**	16,511	9,419	7,092	1,613	803	810
**2015**	17,593	9,997	7,596	1,725	873	852
**2016**	18,045	10,383	7,662	1,697	830	867
**2017**	18,649	10,610	8,039	1,620	809	811
**2018**	20,155	11,373	8,782	1,737	844	893
**2019**	18,722	10,590	8,132	1,663	806	857
**TOTAL**	195,567	111,145	84,422	18,721	9,345	9,376
**MEAN**	16,297	9,262	7,035	1,560	779	781
***SD***	*2,273.1*	*1,292.1*	*984.4*	*157.7*	*75.1*	*86.5*
**MEDIAN**	16,203	9,212	6,991	1,617	805	811
***IQR (Q1)***	*14,533.3*	*8,269.5*	*6,263.8*	*1,471.0*	*769.8*	*715.3*
***IQR (Q3)***	*18,196.0*	*10,434.8*	*7,756.3*	*1,671.5*	*824.0*	*853.3*

**Figure 3:**
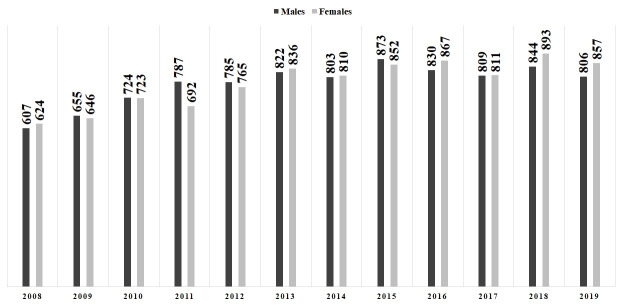
Total number of in-hospital deaths per year in 195,567 urgent hospitalizations due to (I.74) Arterial Thromboses and Embolisms in the Unified Health System in Brazil, by gender (2008-2019).

**Figure 4:**
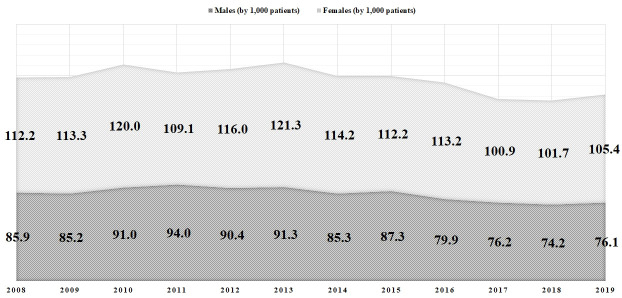
Annual in-hospital mortality rates (per 1,000 patients) in 195,567 urgent hospitalizations due to (I.74) Arterial Thromboses and Embolisms in the Unified Health System in Brazil, by gender (2008-2019).

Eighteen thousand seven hundred and twenty-one deaths (n=18,721) were associated with emergency admissions for ATE in the studied period. Women accounted for 9,376 (50.1%) deaths, with an average of 781 fatalities/year (SD=86.57), and men totaled 9,345 (49.9%) deaths, with an average of 779 fatalities/year (SD=75.17) (Table 1; Figure 3). The average female in-hospital mortality rate (112 deaths per 1,000 cases) was significantly higher than the average male rate (85 deaths per 1,000 cases) (p<0.05) (Figure 4). Moreover, women exhibited a higher risk of death compared to men (OR=1.36; 95%CI=1.32-1.4; p<0.05). Despite these results, overall in-hospital survival exhibited a rising trend from 2008 to 2019 (r^2^=0.9576), with mean growth of 3.9%/year for males (SD=0.04) and 3.7%/year for females (SD=0.04), yet the annual average of surviving patients was significantly higher among men when compared to women (8,483/year versus 6,254/year; p<0.05).

Data from 2008-2019 regarding ethnicity demonstrated that whites were the most prevalent ethnic group, with a total of 86,966 hospital admissions (males=57.8% and females=42.2%), followed by browns (n=47,120 cases; males=56.2% and females=43.8%), blacks (n=8,357 cases; males=55.4% and females=44.6%), and indigenous Brazilians (n=94 cases; males=55.3% and females=44.7%), as seen in Table 2. Whites also accounted for the highest number of fatalities (n=8,067 deaths; males=49.6% and females=50.4%), followed by browns (n=4,326 deaths; males=50.9% and females=49.1%), blacks (n=656 deaths; males=48.8% and females=51.2%), and indigenous Brazilians (n=13 deaths; males=61.5% and females=38.5%) (Table 2). Self-declared black ethnicity was associated with a protective effect in both males (OR=0.86; 95%CI=0.77-0.97; p<0.05) and females (OR=0.79; 95%CI=0.70-0.89; p<0.05) when compared to the self-declared white ethnicity gender-matched groups (Table 3). Brown females were associated with a protective effect when compared to white females (OR=0.90; 95%CI=0.86-0.96; p<0.05). No association was observed among brown males or indigenous Brazilian males and females when compared to whites (Table 3). Compared to men, women exhibited a higher risk of death among whites (OR=1.44; 95%CI=1.4-1.5; p<0.05), blacks (OR=1.33; 95%CI=1.3-1.5; p<0.05), and browns (OR=1.25; 95%CI=1.2-1.3; p<0.05), as showed in Table 4. No association was observed among indigenous Brazilians (p=0.62).

**Table 2:** Number of hospital admissions and in-hospital deaths in 195,567 urgent hospitalizations due to (I.74) Arterial Thromboses and Embolisms in the Unified Health System in Brazil, by gender and ethnicity (2008-2019).

	**HOSPITAL ADMISSIONS**								**IN-HOSPITAL DEATHS**							
	***WHITE***		***BROWN***		***BLACK***		***INDIGENOUS BRAZILIANS***		***WHITE***		***BROWN***		***BLACK***		***INDIGENOUS BRAZILIANS***	
	***M***	***F***	***M***	***F***	***M***	***F***	***M***	***F***	***M***	***F***	***M***	***F***	***M***	***F***	***M***	***F***
**2008**	3,473	2,766	1,056	809	274	205	8	6	247	276	97	82	22	15	3	1
**2009**	3,619	2,607	1,308	1,026	332	267	14	12	302	292	116	88	23	26	3	1
**2010**	3,746	2,814	1,347	1,040	286	247	9	8	313	301	122	135	22	23	2	1
**2011**	3,962	2,965	1,422	1,140	355	263	2	0	336	304	133	104	17	28	0	0
**2012**	3,749	2,850	1,486	1,197	307	270	4	1	324	322	121	130	29	20	0	0
**2013**	4,164	3,029	1,987	1,569	366	272	3	0	351	355	174	165	36	31	0	0
**2014**	4,276	2,990	2,268	1,815	373	326	4	2	351	341	203	207	31	30	0	1
**2015**	4,505	3,255	2,682	2,015	424	339	1	4	356	382	246	207	26	32	0	1
**2016**	4,683	3,327	2,888	2,157	500	366	2	3	354	385	233	240	32	33	0	0
**2017**	4,560	3,300	3,226	2,467	434	362	2	0	350	334	262	241	30	26	0	0
**2018**	4,898	3,512	3,391	2,673	480	394	2	2	365	382	244	240	23	34	0	0
**2019**	4,977	3,517	3,488	2,808	523	426	1	4	376	409	261	283	32	40	0	0
**TOTAL**	50,612	36,932	26,549	20,716	4,654	3,737	52	42	4,025	4,083	2,212	2,122	323	338	8	5
**MEAN**	4,218	3,078	2,212	1,726	388	311	4	4	335	340	184	177	27	28	1	0
***SD***	*490.10*	*286.86*	*857.86*	*667.28*	*80.38*	*64.24*	*3.82*	*3.50*	*33.61*	*40.94*	*61.19*	*65.49*	*5.35*	*6.45*	*1.18*	*0.49*
**MEDIAN**	4,220	3,010	2,128	1,692	370	299	3	3	351	338	189	186	28	29	0	0
***IQR (Q1)***	*3,748.3*	*2,841.0*	*1,403.3*	*1,115.0*	*325.8*	*266.0*	*2.0*	*0.8*	*321.3*	*303.3*	*121.8*	*123.5*	*22.8*	*25.3*	*-*	*-*
***IQR (Q3)***	*4,590.8*	*3,306.8*	*2,972.5*	*2,234.5*	*445.5*	*363.0*	*5.0*	*4.5*	*354.5*	*382.0*	*244.5*	*240.0*	*31.3*	*32.3*	*0.5*	*1.0*

M: Males; F: Females.

**Table 3:** Comparative analysis between ethnicity for odds ratio (OR) of death by sex in 195,567 urgent hospital admissions due to ATE from 2008 to 2019 in the Unified Health System in Brazil.

	**FEMALES**			**MALES**		
	**OR**	**95%CI**	***p-value***	**OR**	**95%CI**	***p-value***
**Whites**	1.0*	-		1.0*	-	
**Blacks**	0.79	0.70-0.89	<0.05	0.86	0.77-0.97	<0.05
**Browns**	0.90	0.86-0.96	<0.05	-	-	N. S.
**Indigenous Brazilians**	-	-	N. S.	-	-	N. S.

* Reference; N. S.: not significant.

**Table 4:** Comparative analysis between genders for odds ratio (OR) of death by ethnicity in 195,567 urgent hospital admissions due to ATE from 2008 to 2019 in the Unified Health System in Brazil.

	**OR**	**95%CI**	***p-value***
**Whites**			
**Male**	1.0*	-	
**Female**	1.44	1.4-1.5	<0.05
**Blacks**			
**Male**	1.0*	-	
**Female**	1.33	1.3-1.5	<0.05
**Browns**			
**Male**	1.0*	-	
**Female**	1.25	1.2-1.3	<0.05
**Indigenous Brazilians**			
**Male**	1.0*	-	
**Female**	-	-	*N. S.*

* Reference; N. S.: not significant.

The age of 50 years represented an important factor for worse outcomes in our analyses (Table 5). The majority of deaths occurred in patients over 50, in both men (total=8,641; 91.9% of all deaths in men; mean=720/year; SD=88.02) and women (total=8,823; 93.7% of all deaths in women; mean=735/year; SD = 98.90). Being over 50 years of age imposed a significantly higher risk of death for both males (OR=2.08; 95%CI=1.9-2.2; p<0.05) and females (OR=3.54; 95%CI=3.2-3.8; p<0.05), while a protective effect was associated with ages under 45 (males: OR=0.51; 95%CI=0.46-0.56; p<0.05; females: OR=0.31; 95%CI=0.28-0.34; p<0.05) and 40 (males: OR=0.55; 95%CI=0.49-0.61; p<0.05; females: OR=0.32; 95%CI=0.28-0.36; p<0.05) for both sexes.

**Table 5:** Hospitalizations and in-hospital deaths in 195,567 urgent hospitalizations due to (I.74) Arterial Thromboses and Embolisms in the Unified Health System in Brazil, by gender and age (2008-2019).

	**MALE**				**FEMALE**			
	**HOSPITAL ADMISSIONS**		**IN-HOSPITAL DEATHS**		**HOSPITAL ADMISSIONS**		**IN-HOSPITAL DEATHS**	
	***UNDER 50 YEARS***	***OVER 50 YEARS***	***UNDER 50 YEARS (HMR%)***	***OVER 50 YEARS (HMR%)***	***UNDER 50 YEARS***	***OVER 50 YEARS***	***UNDER 50 YEARS (HMR%)***	***OVER 50 YEARS (HMR%)***
2008	1,380	5,590	62 (4.5%)	508 (9.1%)	1,115	3,427	38 (3.4%)	548 (16.0%)
2009	1,284	6,332	42 (3.3%)	616 (9.7%)	1,009	3,681	46 (4.6%)	607 (16.5%)
2010	1,402	6,585	80 (5.7%)	659 (10.0%)	1,110	4,918	47 (4.2%)	683 (13.9%)
2011	1,329	6,918	59 (4.4%)	701 (10.1%)	1,125	5,066	47 (4.2%)	627 (12.4%)
2012	1,324	7,355	66 (5.0%)	705 (9.6%)	1,116	5,495	50 (4.5%)	709 (12.9%)
2013	1,342	7,709	73 (5.4%)	771 (10.0%)	1,055	5,844	39 (3.7%)	790 (13.5%)
2014	1,365	7,903	79 (5.8%)	726 (9.2%)	1,119	4,504	41 (3.7%)	777 (17.3%)
2015	1,358	8,774	67 (4.9%)	822 (9.4%)	1,254	4,939	68 (5.4%)	789 (16.0%)
2016	1,440	8,951	64 (4.4%)	765 (8.5%)	1,249	6,506	52 (4.2%)	853 (13.1%)
2017	1,371	9,146	56 (4.1%)	744 (8.1%)	1,280	6,662	51 (4.0%)	735 (11.0%)
2018	1,408	9,846	57 (4.0%)	799 (8.1%)	1,337	7,319	53 (4.0%)	823 (11.2%)
2019	1,484	10,246	47 (3.2%)	825 (8.1%)	1,478	7,527	63 (4.3%)	882 (11.7%)
TOTAL	16,487	95,355	752 (4.6%)	8,641 (9.1%)	14,247	65,888	595 (4.2%)	8,823 (13.4%)
MEAN	1,374	7,946	63	720	1,187	5,491	50	735
*SD*	*52.0*	*1399.6*	*11.1*	*88.0*	*128.4*	*1,269.4*	*8.6*	*98.9*
MEDIAN	1,368	7,806	63	735	1,122	5,281	49	756
*IQR (Q1)*	*1,338.8*	*6834.8*	*56.8*	*690.5*	*1,113.8*	*4,814.5*	*44.8*	*669.0*
*IQR (Q3)*	*1,403.5*	*8999.8*	*68.5*	*778.0*	*1,260.5*	*6,545.0*	*52.3*	*798.3*

HMR: In-Hospital Mortality Rate.

Using the age cutoff of 50 years as a reference, it was observed that the effect of aging, by comparing with consecutive five-year age-matched groups, increased the risk of death for both sexes, yet the risk increment was more substantial in women, as seen in Table 6. Moreover, when comparing sexes and consecutive five-year age-matched groups, women persistently exhibited higher risk of death, as demonstrated in Table 7. Despite not attaining statistical significance, females in age groups between 40 and 50 years tended to be associated with a possible protective effect, even if only partially (Table 7).

**Table 6:** Comparative analysis between age groups for odds ratio (OR) of death by sex in 195,567 urgent hospital admissions due to ATE from 2008 to 2019 in the Unified Health System in Brazil.

	**FEMALES**			**MALES**				
	**OR**	**95%CI**	***p-value***	**OR**	**95%CI**	***p-value***		
**Patients under 49 years of age**	1.0*	-		1.0*	-		
**Patients aged 50 years or more**	3.54	3.2-3.8	<0.05	2.08	1.9-2.2	<0.05
**Patients aged 55 years or more**	3.78	3.4-4.1	<0.05	2.20	2.0-2.4	<0.05
**Patients aged 60 years or more**	4.10	3.7-4.4	<0.05	2.40	2.2-2.6	<0.05
**Patients aged 65 years or more**	4.57	4.2-5.0	<0.05	2.67	2.5-2.9	<0.05
**Patients aged 70 years or more**	5.30	4.8-5.8	<0.05	3.07	2.8-3.3	<0.05
**Patients aged 75 years or more**	6.53	5.9-7.1	<0.05	3.59	3.3-3.9	<0.05

* Reference.

**Table 7:** Comparative analysis between genders for odds ratio (OR) of death by age groups in 195,567 urgent hospital admissions due to ATE from 2008 to 2019 in the Unified Health System in Brazil.

	**OR**	**95%CI**	***p-value***
**Patients under 40 years of age**			
**Male**	1.0*	-	
**Female**	0.86	0.7-1.01	0.077
**Patients under 45 years of age**			
**Male**	1.0*	-	
**Female**	0.91	0.8-1.04	0.174
**Patients under 50 years of age**			
**Male**	1.0*	-	
**Female**	0.91	0.8-1.01	0.100
**Patients over 50 years of age**			
**Male**	1.0*	-	
**Female**	1.55	1.5-1.6	<0.05
**Patients over 55 years of age**			
**Male**	1.0*	-	
**Female**	1.56	1.5-1.6	<0.05
**Patients over 60 years of age**			
**Male**	1.0*	-	
**Female**	1.56	1.5-1.6	<0.05
**Patients over 65 years of age**			
**Male**	1.0*	-	
**Female**	1.56	1.5-1.6	<0.05
**Patients over 70 years of age**			
**Male**	1.0*	-	
**Female**	1.57	1.5-1.6	<0.05
**Patients over 75 years of age**			
**Male**	1.0*	-	
**Female**	1.65	1.6-1.7	<0.05

* Reference.

## DISCUSSION

Peripheral arterial occlusive disease affects more than 200 million people worldwide and in Brazil ALI is responsible for more than 12,000 urgent hospitalizations every year.^10^^,^^14^^,^^15^ In this regard, our analyses revealed a rising trend in overall number of hospital admissions on an emergency basis due to arterial occlusions (r^2^=0.9584; growth rate=3.7%/year) and the results also showed an improvement in overall in-hospital survival over the years 2008 to 2019, which, in turn, might reflect the contribution of developments in vascular surgery and endovascular procedures in public health nationwide, expansion of emergency services in the SUS, the increasing awareness of vascular diseases and the rising proportion of elderly people in the Brazilian population.

In our study, we observed that gender is a relevant risk factor associated with emergencies due to ATE that might influence the outcomes of ALI. Even though Brazilian men presented a significantly higher annual average number of emergency hospital admissions, risks of death due to emergency ATE remained remarkably higher among Brazilian women. Previous reports have suggested a significant correlation between worse outcomes and deaths from arterial occlusions with female sex.^3^^,^^9^^,^^10^^,^^14^^,^^16^^,^^17^ Nevertheless, the causes of the surprisingly higher in-hospital mortality rates of ALI in women are not fully understood.

In the United States (US), the Life Line Screening program studied 133,750 women and 71,996 men who shared similar risk factors for vascular diseases, demonstrating that women were more likely to present findings of occlusive arterial disease in the physical examination.^3^^,^^4^ Furthermore, Egorova analyzed 2.4 million PAD-related inpatient records in the US, from 1998 to 2007, and showed that women had a higher intervention-related mortality rate compared to men, that mortality rates in females were persistently higher even after surgical procedures, and also associated women with a longer length of stay.^18^ Contradictorily, our data showed no difference in the length of stay between sexes admitted on an emergency basis for care of ALI.

Lejay evaluated 269 women and 315 men with critical limb ischemia, showing that female gender was an independent factor predicting death, even with adjustment for age.^19^ One result of our investigation was that Brazilian women were associated with a higher in-hospital mortality rate (112/1,000 cases versus 85/1,000 cases; p<0.05) and a higher chance of death (OR=1.36; p<0.05) due to emergency arterial occlusions.

Numerous studies have suggested that these sex-related discrepancies might be related to differences in patterns of presentation, clinical features, and responses to treatments, including the Brazilian study by Nascimento et al., which showed worse surgical outcomes and higher mortality among women undergoing surgical and endovascular revascularization for treatment of arterial disease.^20^ The more severe and complex lesions with which women present may be a consequence of numerous factors, such as smaller vessel diameter, higher rates of multilevel disease, and smaller calf muscle mass.^20^^,^^21^ In social and behavioral aspects, previous reports have recognized that women suffering from ALI are usually at older ages, socially isolated, and have lower income, which may hinder access to specialized medical care, and they also might be caregivers of old and sick husbands, which frequently may lead them to neglect and/or underestimate their own medical needs, and, in turn, might also explain the older peak age regarding hospitalization among women in our data.^18^^,^^20^

The delayed detection of ALI in females might be a result of misdiagnosis of the symptoms of early arterial insufficiency with other frequent disorders with similar clinical patterns in older women, such as arthritis.^18^ In our data, the peak age for urgent hospitalization in women was considerably more advanced than in men (80 years or more versus 65 to 69 years), as previously mentioned, which also possibly influences the higher mortality rate associated with females. Our age analyses revealed that women persistently exhibited higher risk of death in all groups over the age of 50. This indicates that the effect of aging, despite imposing a higher risk of death in both groups, seems to affect women more severely when compared to men. Moreover, although not statistically confirmed, our results suggested that women under 50 years of age seem to be associated with some degree of protective effect compared to men. The literature attributes these protective vascular effects to the endogenous estrogen levels of women in the menacme, usually under 50 years of age.^21^^,^^22^

Several cross-sectional studies, randomized controlled trials, and systematic reviews have reported some degree of evidence that sex hormones may influence the course of ALI, PAD, atherosclerosis, and metabolic syndrome.^11^ Estrogen plays a relevant role in vascular physiology, by upregulating nitric oxide synthase, stimulating arterial smooth muscle vasodilation, enhancing endothelial cell vasoreactivity, reducing oxidative stress, and protecting from lipid oxidation and vascular injury.^21^ Thus, estrogen presents beneficial effects on vascular lipid metabolism and metabolic syndrome, which, in turn, protects from endothelial injury – a known hallmark that triggers the cascade of events leading to arterial occlusions.^21^^,^^22^

Besides, we cannot disregard that arterial disease is a systemic condition and limb ischemia is usually a late-stage manifestation of vascular disease, and individuals suffering PAD events are more likely to present systemic atherosclerosis affecting other vascular beds.^23^ Several authors suggest that men are more likely to manifest further atherothrombotic complications like angina, myocardial infarction, stroke, and death, hence, women might have more ALI complications because men die earlier from coronary and cerebrovascular disease before developing complications from PAD.^4^^,^^8^^,^^15^^,^^17^^,^^23^^,^^24^

The epidemiology of PAD seems also to be influenced by ethnicity and varies by race. The prevalence of ALI is higher among afro-descendants than whites and Hispanics, and blacks are also associated with worse outcomes.^14^^,^^25^ Collins reported, in a 403-patient study in the US, that the prevalence of ALI was higher among African-Americans (46.2%), followed by Hispanics (26.9%) and whites (26.9%).^25^ Rowe assessed 87,337 patients admitted due for emergency ALI in the US and associated higher risk of amputations with blacks and Hispanics, whereas whites were more likely to undergo revascularization.^26^

Contradictorily, our results revealed that the highest annual mean hospitalizations and deaths were recorded for whites, for both males and females, and, moreover, blacks of both sexes and brown females were associated with a protective effect when compared to self-declared Caucasians. A report from Brazil, part of the Baependi Heart Study in the state of Minas Gerais that assessed the occurrence of ALI in a Brazilian rural population, also showed a higher prevalence of ALI in blacks compared to whites.^27^ It is important to recognize that the high prevalence among whites in our results might suggest bias in the actual prevalence and risk compared to dark-skinned ethnic groups since black people experience less access to medical care due to economic disparities in Brazil.

To date, there is no previous nationwide study that statistically compared urgent cases of ALI and risks across gender and ethnic groups in Brazil. Furthering this challenge, our results provide evidence that, compared to men, Brazilian women presented a higher chance of death across the three major ethnic groups (whites, blacks, and browns). In the Multi-Ethnic Study of Atherosclerosis (MESA), Forbang reported that the prevalence of ALI was higher among women of all ethnic groups.^28^ It is important to emphasize that race-gender stratification may predict outcomes that may not be properly evaluated by gender or race alone, since social inequality issues in Brazil need to be taken into considerations, as already mentioned in this discussion.

Our study had several limitations. First, the limitation of its study design and the database (retrospective and observational study with secondary data from DATASUS), the nature of which did not allow us to discriminate the type of treatment/intervention, to evaluate the prognosis and recovery after the procedures, or to differentiate between the proportions of thrombosis and embolism. Also, there is the possibility that patients with chronic ischemia who had become acute were urgently hospitalized under the I.74 code, which would represent an analytical confounder, since it is not feasible to separate acute arterial occlusions from chronic arterial occlusions that have become acute. Another point of concern is the possibility of the increasing tendency towards outpatient procedures in Brazil, such as endovascular interventions on an outpatient practice basis (ambulatory procedures), which are not counted in this database. Besides, it was not possible to evaluate the clinical and laboratory characteristics of patients at the time of hospitalization, such as comorbidities, diagnosis of metabolic syndrome, history of vascular interventions, impairment of daily activities, Rutherford class, body mass index and obesity, smoking habit, alcohol consumption, the number of readmissions of patients by gender, laboratory or clinical diagnosis of menopause, or evaluation of laboratory biomarkers. It is also pertinent to recognize that the figures in the DATASUS database are constantly being updated (as stated in footnotes on the platform itself), and minimal differences may appear from time to time. All these variables might constitute residual or unmeasured confounding factors that could have interfered with the analysis, which, in turn, constitutes grounds for conducting more robust studies.

## CONCLUSION

This study poses females at greater risk for in-hospital mortality associated with urgent vascular hospitalizations, independently of race and age. In the current scenario of emergency admissions due to ALI, Brazilian women die more, while Brazilian men survive more, regardless of ethnicity and age. Since women in Brazil experience worse outcomes, it is imperative that more detailed and larger studies be performed, helping to better understand the nuances of gender-specific prevalence, associated factors, and how they impact the Brazilian population suffering from ALI.
